# Supplementary material for: Large-Scale Field Trials of an Eimeria Vaccine Induce Positive Effects on the Production Index of Broilers
Source: Vaccines (Basel). 2024 Jul 19;12(7):800. doi: 10.3390/vaccines12070800 (PMC11281675; doi:10.3390/vaccines12070800)
Supplement: Supplementary file 1 [file vaccines-12-00800-s001.zip › vaccines-3090856 - Supplementary Figures (1 and 2) v2.pptx]

## Slide 1
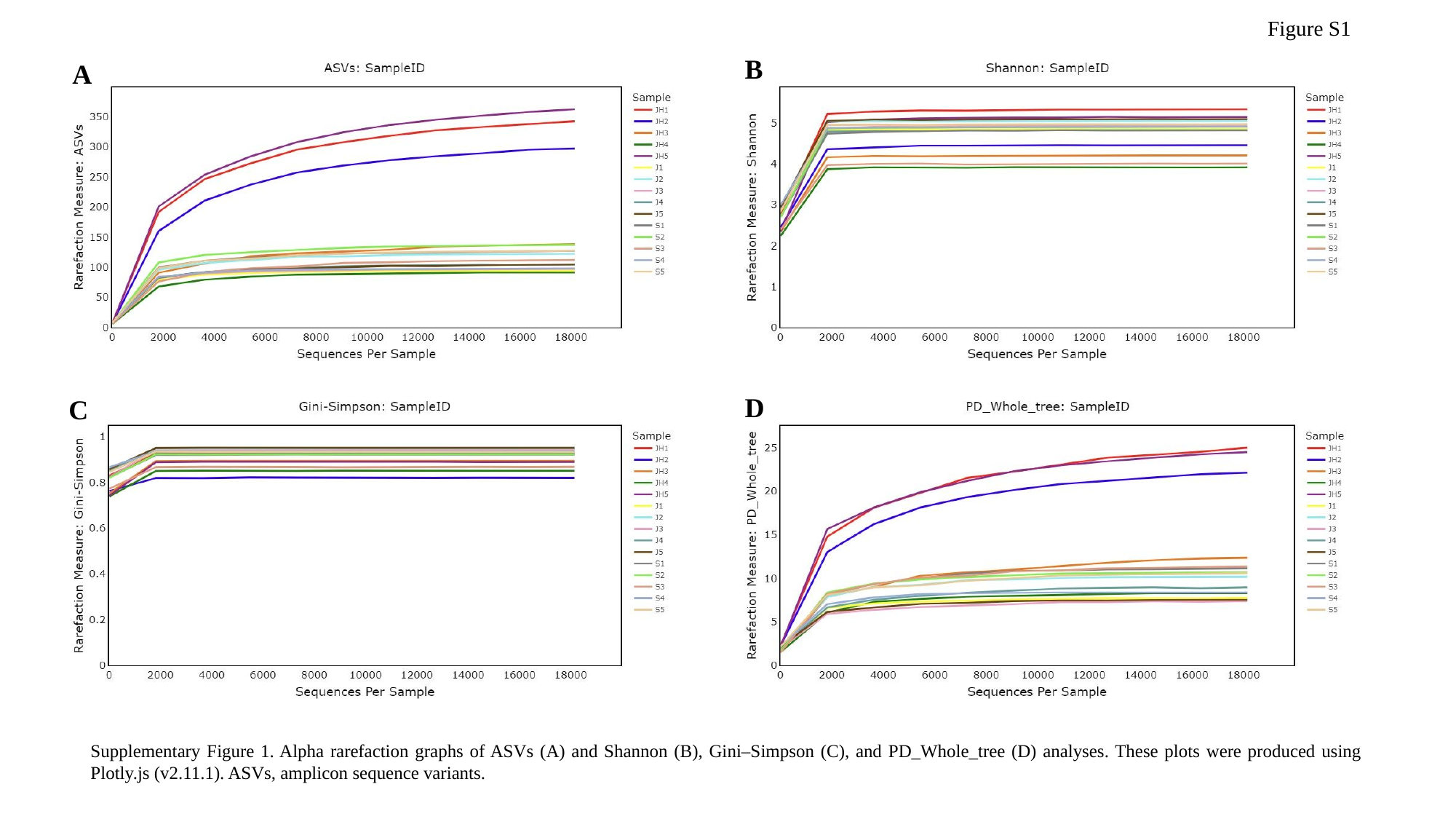

Figure S1
B
A
D
C
Supplementary Figure 1. Alpha rarefaction graphs of ASVs (A) and Shannon (B), Gini–Simpson (C), and PD_Whole_tree (D) analyses. These plots were produced using Plotly.js (v2.11.1). ASVs, amplicon sequence variants.

## Slide 2
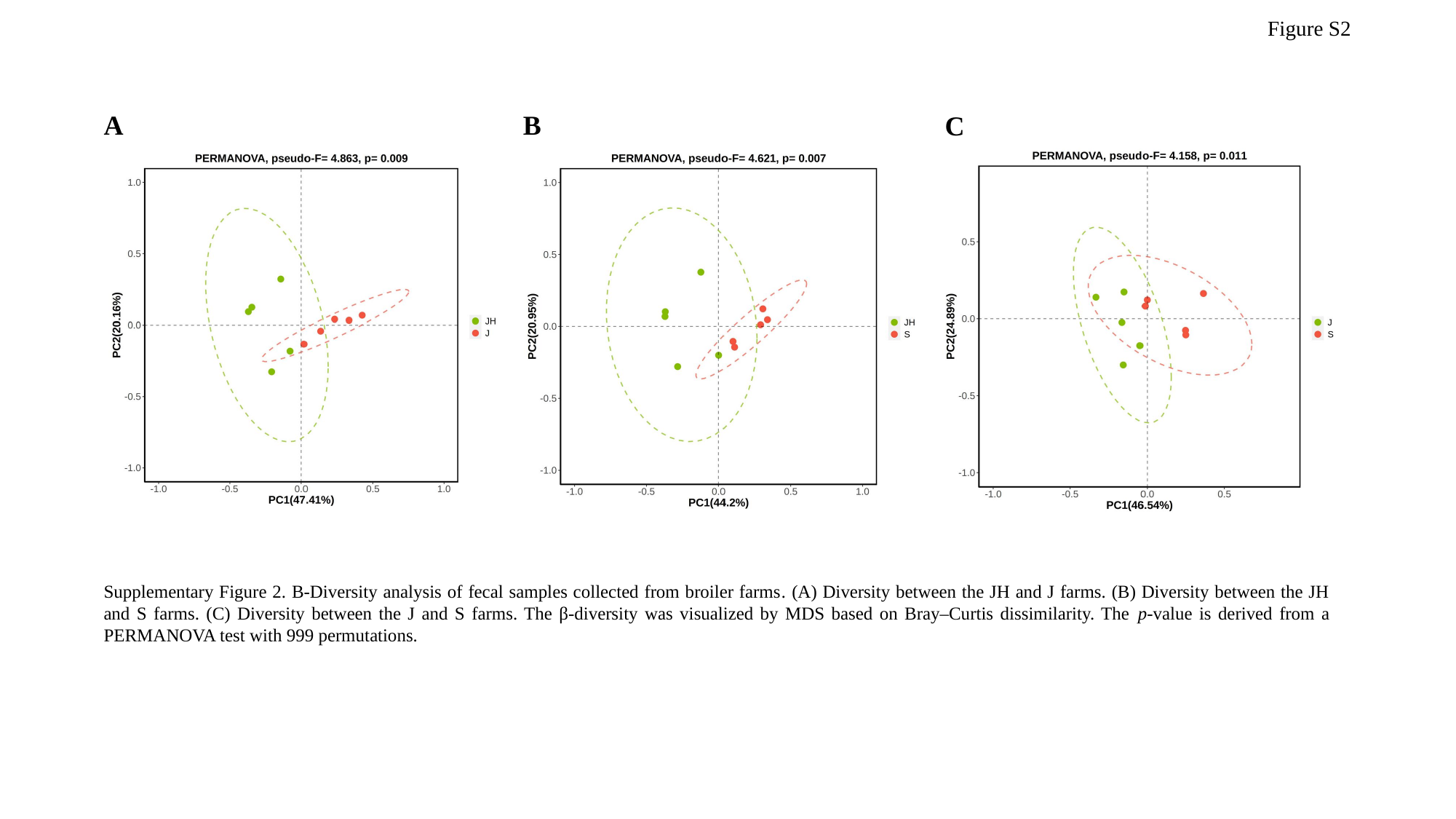

Figure S2
A
B
C
Supplementary Figure 2. Β-Diversity analysis of fecal samples collected from broiler farms. (A) Diversity between the JH and J farms. (B) Diversity between the JH and S farms. (C) Diversity between the J and S farms. The β-diversity was visualized by MDS based on Bray–Curtis dissimilarity. The p-value is derived from a PERMANOVA test with 999 permutations.
